# Validation of an ICD-9-CM-Based Monitoring Tool for Regional Trauma Systems: The PaTraME Study in Pavia Province, Italy

**DOI:** 10.3390/medsci14010013

**Published:** 2025-12-27

**Authors:** Paola Fugazzola, Leandro Gentile, Francesco Chiarolanza, Pietro Perotti, Mario Alessiani, Federico Capra Marzani, Lorenzo Cobianchi, Simone Frassini, Federico Alberto Grassi, Catherine Klersy, Alba Muzzi, Alessandra Palo, Stefano Perlini, Maurizio Raimondi, Luca Ansaloni, on behalf of the PaTraME Study Group

**Affiliations:** 1 General Surgery, Fondazione IRCCS (Istituto di Ricovero e Cura a Carattere Scientifico) Policlinico San Matteo, 27100 Pavia, Italy; paola.fugazzola@smatteo.pv.it (P.F.); l.cobianchi@smatteo.pv.it (L.C.); simone.frassini01@universitadipavia.it (S.F.); 2 Dipartimento di Scienze Clinico-Chirurgiche, Diagnostiche e Pediatriche, University of Pavia, 27100 Pavia, Italy; francesco.chiarolanza01@universitadipavia.it (F.C.); mario.alessiani@unipv.it (M.A.); federico.grassi@unipv.it (F.A.G.); 3 Department of Public Health, Experimental and Forensic Medicine, University of Pavia, 27100 Pavia, Italy; l.gentile@smatteo.pv.it; 4 UOC (Unità Operativa Complessa) Osservatorio Epidemiologico per il Governo della Domanda, ATS (Agenzia di Tutela della Salute) Pavia, 27100 Pavia, Italy; pietro_perotti@ats-pavia.it; 5 UOC (Unità Operativa Complessa) Chirurgia Generale, Ospedale Unificato di Broni-Stradella, ASST (Azienda Socio Sanitaria Territoneiale) Pavia, 2049 Pavia, Italy; 6 UOC (Unità Operativa Complessa) Anestesia e Rianimazione 1, Fondazione IRCCS (Istituto di Ricovero e Cura a Carattere Scientifico) Policlinico San Matteo, 27100 Pavia, Italy; f.capra@smatteo.pv.it; 7 UOC (Unità Operativa Complessa) Ortopedia e Traumatologia, Fondazione IRCCS (Istituto di Ricovero e Cura a Carattere Scientifico) Policlinico San Matteo, 27100 Pavia, Italy; 8 Biostatistics, Epidemiology & Research Design (BERD) Unit, Research Department Fondazione IRCCS (Istituto di Ricovero e Cura a Carattere Scientifico) Policlinico San Matteo, 27100 Pavia, Italy; c.klersy@smatteo.pv.it; 9 Direzione Sanitaria, Fondazione IRCCS (Istituto di Ricovero e Cura a Carattere Scientifico) Policlinico San Matteo, 27100 Pavia, Italy; a.muzzi@smatteo.pv.it; 10 UOSD (Unità Operativa Semplice di Dipartimento) AAT (Articolazioni Territoriali Agenzia) di Pavia, Fondazione IRCCS (Istituto di Ricovero e Cura a Carattere Scientifico) Policlinico San Matteo, 27100 Pavia, Italy; a.palo@smatteo.pv.it; 11 UOC (Unità Operativa Complessa) Medicina di Urgenza, Fondazione IRCCS (Istituto di Ricovero e Cura a Carattere Scientifico) Policlinico San Matteo, 27100 Pavia, Italy; s.perlini@smatteo.pv.it; 12 Dipartimento di Medicina Interna e Terapia Medica, University of Pavia, 27100 Pavia, Italy; 13 UOC (Unità Operativa Complessa) Anestesia e Rianimazione Oltrepò, ASST (Azienda Socio Sanitaria Territoneiale) Pavia, 27100 Pavia, Italy; maurizio_raimondi@asst-pavia.it

**Keywords:** trauma system, centralization, administrative data, TMPM-ICD9, XISS, quality monitoring

## Abstract

**Background/Objectives**: Continuous trauma-system monitoring is limited by the lack of scalable, low-cost tools. The Pavia Trauma Management Epidemiology (PaTraME) project uses routinely collected ICD-9-CM discharge data (SDO) and the Trauma Mortality Probability Model (TMPM) to derive Injury Severity Score (XISS) and probability of death (TMPM-POD), creating a cost-free surveillance framework for regional trauma networks. **Methods**: We conducted a retrospective study of all major-trauma admissions (XISS > 15) in Pavia Province from 2014 to 2021. Anonymized SDO records were linked with emergency department flows and mortality registries. XISS and TMPM-POD were computed for each case. Case volumes, severity distributions, hub-centralization, and mortality (in-hospital, 30-day, and 180-day) were analyzed using trend and regression models (*p* < 0.05). **Conclusions**: We identified 1959 major-trauma admissions. Volumes increased up to 2019, dropped during the COVID-19 pandemic, and partially recovered in 2021 (*p* < 0.001). Overall, 61.5% of patients were admitted to hub centers, with an upward trend (*p* < 0.001). Hubs treated more severe trauma (median XISS 17 vs. 16; TMPM-POD 0.06 vs. 0.05, both *p* < 0.001). In-hospital mortality remained stable (8.2–11.4%, *p* = 0.828). TMPM-POD showed strong agreement with observed in-hospital mortality (Lin’s concordance correlation coefficient 0.81), though calibration worsened at higher risk levels. PaTraME confirms TMPM-POD as a valid mortality predictor and demonstrates a reproducible administrative-data framework for trauma surveillance. Rising hub admissions and stable mortality despite increasing complexity suggest improved system performance. Stratification of XISS and TMPM-POD between hub and spoke centers highlights peripheral hospitals managing disproportionately severe cases, informing targeted resource allocation and supporting quality improvement via automated dashboards.

## 1. Introduction

Trauma remains a leading cause of death and disability, necessitating efficient prehospital triage, centralized care at specialized hubs, and ongoing quality monitoring [1,2]. Traditional trauma registries provide detailed data but are resource-intensive and often limited in geographic scope. Administrative discharge data (SDO) coded by ICD-9-CM, combined with empirically derived severity models such as TMPM-ICD9 [3], offer a scalable alternative for continuous surveillance of system epidemiology, severity, and centralization performance [4,5,6].

**Figure 1:**
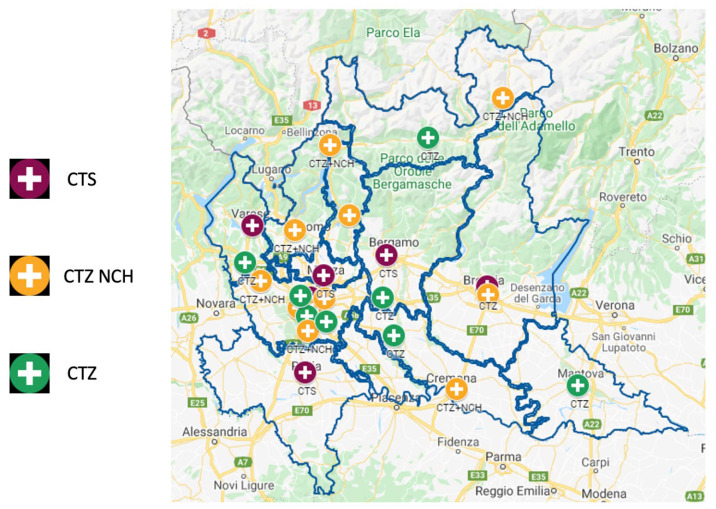
Organization of the regional integrated system for the assistance of the patient suffering from major trauma in Lombardy, Italy.

**Figure 2:**
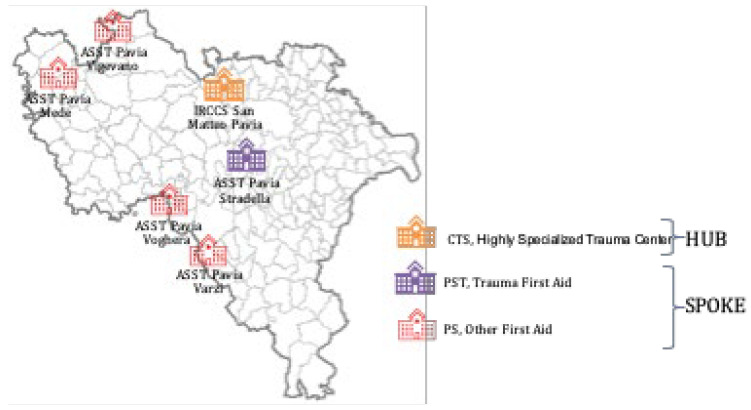
Organization of the regional integrated system for the assistance of the patient suffering from major trauma in the province of Pavia.

The Lombardy trauma network was established by Regional Decree no. 8531 (1 October 2012), which defined the organization of an integrated system for the management of major trauma [7]. The decree was based on a technical document drafted by a group of specialists (GAT, Technical Study Group) from various hospitals. The GAT identified the levels of competence, technology, and professional expertise required for each center according to trauma severity in order to ensure appropriate care. Hospitals were therefore classified into CTS (Highly Specialized Trauma Center), CTZ (Zone Trauma Centre), and PST (Trauma First Aid). With this organizational framework, called Sistema Integrato Assistenza Traumi (SIAT, Integrated System for Trauma Patient Care), the IRCCS San Matteo Hospital Foundation of Pavia has, since 2012, been designated as one of the six first-level trauma centers in Lombardy (CTS, Figure 1) and the only one in Pavia Province. At the time of the analysis, the province included one CTS, no CTZs, and some PSTs (Figure 2).

Quantitative analysis of centralization trends and qualitative assessment of outcomes over time are essential to gauge system maturation and quality improvement. The PaTraME study applies STATA-generated XISS and TMPM-POD [8,9] to all SDO records of traumatic patients in Pavia Province (2014–2021), aiming to validate this toolset for real-time monitoring of trauma system performance and centralization efficiency.

## 2. Materials and Methods

### 2.1. Study Design and Setting

This is a retrospective, population-based observational study of all major-trauma admissions in Pavia Province, Northern Italy over an eight-year period (1 January 2014–31 December 2021). The local trauma network follows the 2012 Lombardy Regional Decree that established the integrated Trauma Care System—hub-and-spoke system—(Sistema Integrato Assistenza Traumi, SIAT) [7], with the IRCCS Policlinico San Matteo as the sole Level I hub and multiple spoke Emergency Departments.

The study protocol was reviewed and approved by the Ethics Committee of the IRCCS Policlinico San Matteo, Pavia (protocol code N.5/D.G./1365, date of approval 21 October 2022). Informed Consent Statement: Given the retrospective nature of the study and the anonymisation of the data, there is no provision for informed consent for participants.

### 2.2. Aim

This project aims to describe access to care for major trauma patients, within SIAT, ten years after its establishment, and assess whether facilities availability influenced trauma patients’ distribution, centralization efficacy, and their overall clinical outcomes (especially mortality rate). The secondary aim of this PaTraME study, which applies STATA-generated XISS [8,9] and TMPM-POD to all SDO records of trauma patients in Pavia Province, is to validate this toolset for real-time monitoring of trauma system performance and centralization efficiency.

### 2.3. Data Sources

Administrative hospital discharge records, including ICD-9-CM diagnosis codes, of all trauma patients (that report one of these diagnoses in any position: 800.xx–904.xx or 920.xx–939.xx or 950.xx–959.xx) admitted in hospitals of Pavia Province from January 2014 to December 2021 have been obtained in collaboration with Provincial Epidemiological Observatory of Local Health Unit (UOC—Osservatorio epidemiologico per il governo della domanda, ATS Pavia). As one traumatic patient could have accessed more than one hospital in cases of secondary centralization or transfers between hospitals, only the access during which the primary treatment was carried out has been included. Transfers occurring within the provincial trauma network were handled by retaining the episode in which primary treatment was delivered and, when available, recording secondary transfer to the hub. Transfers to hospitals outside Pavia Province could not be tracked because extra-provincial discharge episodes were not available in the ATS Pavia dataset; therefore, centralization estimates refer to the within-province network. The XISS, the indirect indicator of Injury Severity Score (ISS), and the TMPM-POD (probability of death) has been calculated for each patient from ICD-9-CM [10,11] codes by the statistical software STATA (release 19.5, StataCorp, College Station, TX, USA) based on the Trauma Mortality Prediction Model created by Osler and Glance [3]. Only patients with a XISS higher than 15, as an indicator of major trauma, have been included in the present study. We used these ICD-9-CM ranges as a screening strategy to maximize sensitivity of case capture from administrative data. The final analytic cohort was defined by XISS > 15. Code groups not representing traumatic injury (e.g., superficial injuries 910–919, burns 940–949/948, and poisoning 960–989) were not included because they fall outside the screening ranges. Foreign-body codes (930–939) are contained within the 920–939 screening block; nevertheless, isolated foreign-body events are unlikely to reach XISS > 15, as XISS is diagnosis-based and requires sufficient anatomic injury burden.

Data about age, sex, date of trauma, admitting hospital, patient arrival modality (118 vs. self-presenting), date of admission, date of discharge, admission ward, discharge ward, discharge modality, diagnosis, procedures, in-hospital and 30-days mortality, and DRG have been collected

In particular, we obtained anonymized individual-level data from three ATS Pavia registries:

1. SDO (Hospital Discharge Records): all hospital episodes with any ICD-9-CM code in the trauma ranges (800.xx–904.xx, 920.xx–939.xx, 950.xx–959.xx).
2. Emergency Room flow (PS flow): ED visits linked to subsequent SDOs, including arrival mode (118 ambulance, self-presenting, helicopter), triage code, and primary complaint.
3. Mortality registry: date and cause of death up to 180 days post-admission.

### 2.4. Variables Collected

For each included patient we extracted the following:

- Demographics: anonymous patient ID, sex, date of birth, and residence.
- Admission details: date/time of ED arrival and hospital admission, hospital ID, ward of entry and discharge, and mode of transport.
- Clinical codes: all ICD-9-CM diagnoses (up to five), procedures (up to five), and DRG.
- Transfers: intra-hospital transfers and secondary transfers to hub (date/time).
- Outcomes: in-hospital death and vital status at 30 days and 180 days post-admission.

### 2.5. Severity Scoring

Using the TMPM module in Stata, we automatically translated ICD-9-CM codes into the following:

- XISS: an indirect Injury Severity Score analogous to AIS-derived ISS.
- TMPM-POD: the Trauma Mortality Probability Model.

### 2.6. Statistical Analysis

Data were analyzed using the Stata software (release 19.5, StataCorp, College Station, TX, USA). A 2-sided *p*-value < 0.05 was considered statistically significant. The Bonferroni correction was used for post hoc comparisons.

Continuous data were described with the median and 25th–75th percentiles, categorical data as counts and percent. The Mann–Whitney U test was used to compare severity scores (XISS and POD) by center type (HUB vs. SPOKE).

For system-level trend analyses, individual-level records were collapsed into aggregated strata (year × center type; and XISS strata), computing stratum size (*N*), mean TMPM-POD, and observed mortality proportion. Subsequent analyses on collapsed datasets used frequency weights equal to N, ensuring that each stratum contributed proportionally to the number of underlying patients and preventing small strata from having the same influence as large strata. This aggregation was adopted to support transparent benchmarking of yearly trends and risk strata using rate-based outcomes, while retaining the appropriate contribution of each stratum through frequency weighting. A limitation of this approach is that aggregation reduces within-stratum variability and individual-level granularity; therefore, estimates may be less informative for very small strata and should be interpreted accordingly. The Cochran–Armitage test for trend was used to compare trends over years between center type. A test for departure from linearity was also performed. Annual admission volumes were modeled using negative binomial regression because volume counts may exhibit overdispersion, violating the Poisson equidispersion assumption. This choice reduces the risk of underestimated standard errors and overly narrow confidence intervals when variance exceeds the mean. Incidence rate ratios (IRRs) with respect to the previous year and 95% confidence intervals (95%CI) were computed. Effect modification of centralization on annual volumes was assessed by including a term of interaction in the model. Annual mortality rates over time were modeled using a binomial generalized regression model with computation of risk differences and 95%CI. Agreement between predicted and observed mortality rates across XISS strata was assessed using Lin’s concordance correlation coefficient and Bland–Altman analysis. CCC was chosen because it captures both correlation and systematic deviation from the line of identity, which is aligned with benchmarking of aggregated rates.

## 3. Results

### 3.1. Epidemiology and Case Volumes

From 2014 to 2021, 1959 major-trauma admissions (XISS > 15) were identified. About half of the patients were male, with a median age of 77 years (Table 1).

**Figure 3:**
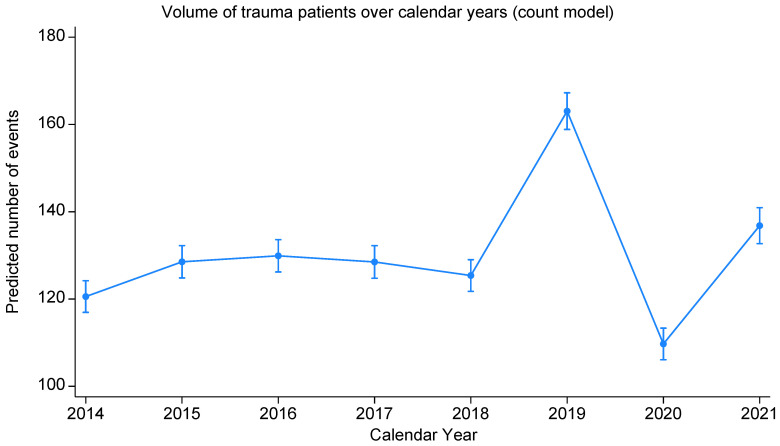
Volume of trauma patients over calendar year (negative binomial regression model).

Case volume rose from 232 in 2014 to a peak of 304 in 2019, dipped to 195 in 2020 during the COVID-19 pandemic, and rebounded to 228 in 2021 (Figure 3), with a significant change over time (bivariable negative binomial regression, *p* < 0.001; Table 2). In particular, 2019 yielded an IRR of 1.25 (95% CI 1.23–1.28) versus 2018, 2020 versus 2019 an IRR of 0.66 (95%CI 0.64–0.68), and 2021 versus 2020 an IRR of 1.22 (95% CI 1.19–1.26). Some departure from linearity was observed both graphically and based on the statistical test (*p* = 0.060).

Centre type was independently associated with trauma volume as well (IRR 1.56, 95%CI 1.54–1.57).

### 3.2. Centralization Trends and System Quality

**Figure 4:**
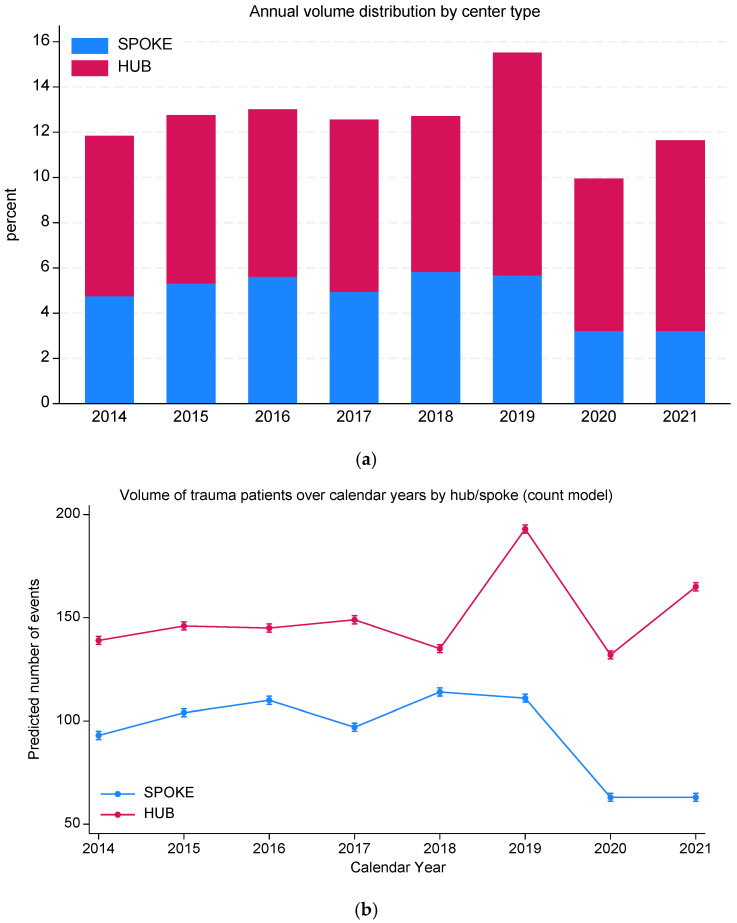
(**a**) Volume of trauma patients over calendar year and centralization pattern (percent within center type); (**b**) volume of trauma patients over calendar year and centralization pattern (negative bi-nomial regression model predicted counts).

Overall, 1204 (61.5%) trauma patients were centralized to hubs. Centralization rates improved significantly over time, from 59.9% in 2014 to 72.4% in 2021 (test for trend, *p* < 0.001; Figure 4a, Table 2), with more recent years associated with higher centralization, despite some fluctuations. Moreover, we observed a significant interaction (*p* < 0.001) between year and centralization, with a progressive decline in spoke volumes compared to a small but progressive increase in hub centers admissions (Figure 4b).

### 3.3. Severity-Score Distributions

**Figure 5:**
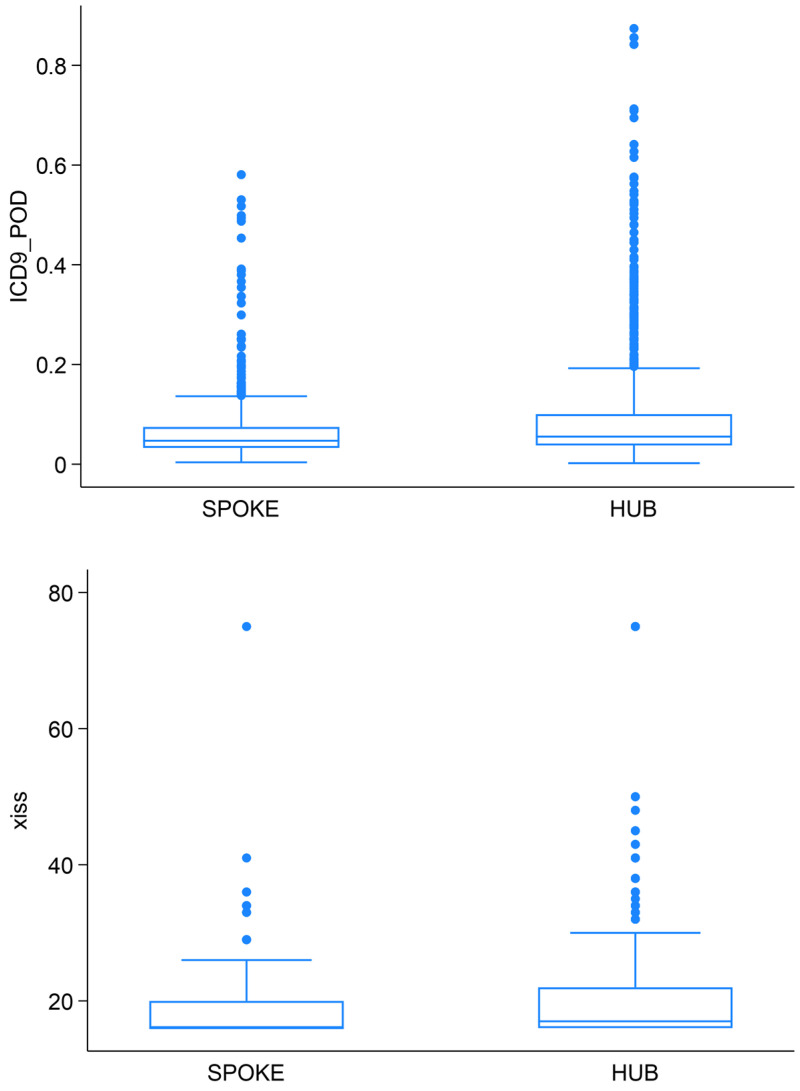
Severity score distribution between spoke and hub.

Median XISS and TMPM-POD were significantly higher in hub centers (XISS: 17 [IQR 16–22]; TMPM-POD: 0.06 [IQR 0.04–0.10]) compared to spokes (XISS: 16 [IQR 16–20]; TMPM-POD: 0.05 [IQR 0.03–0.07]) (Mann–Whitney U test *p* < 0.001 for both, Figure 5).

### 3.4. Mortality Overview

**Figure 6:**
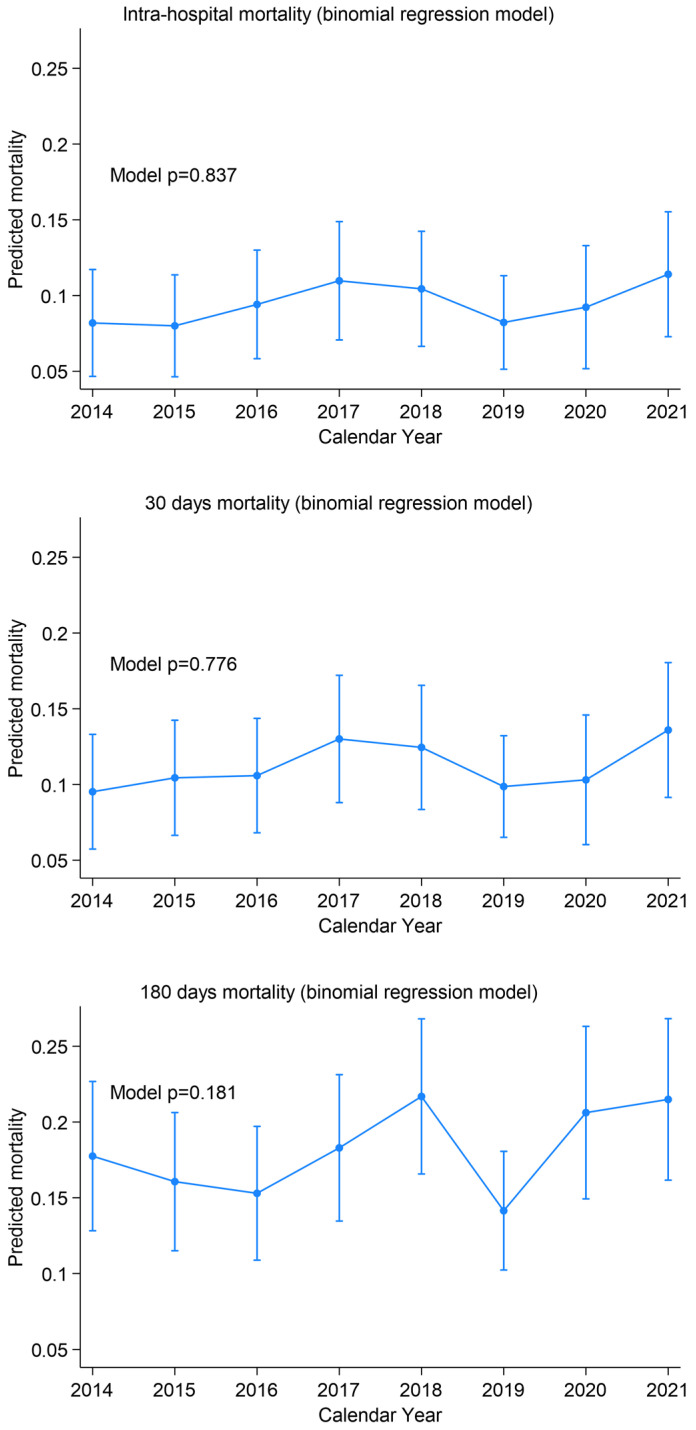
Mortality trends over time.

Global mortality did not change significantly over the study time. In-hospital mortality ranged from 8.2% to 11.4% (test for trend *p* = 0.828, binomial model *p* = 0.837), 30-day mortality from 9.5% to 13.6% (*p* = 0.767 and *p* = 0.776), and 180-day mortality from 17.7% to 21.5% (*p* = 0.184 and *p* = 0.181) (Figure 6).

### 3.5. Correlation Between TMPM-POD and In-Hospital Mortality

**Figure 7:**
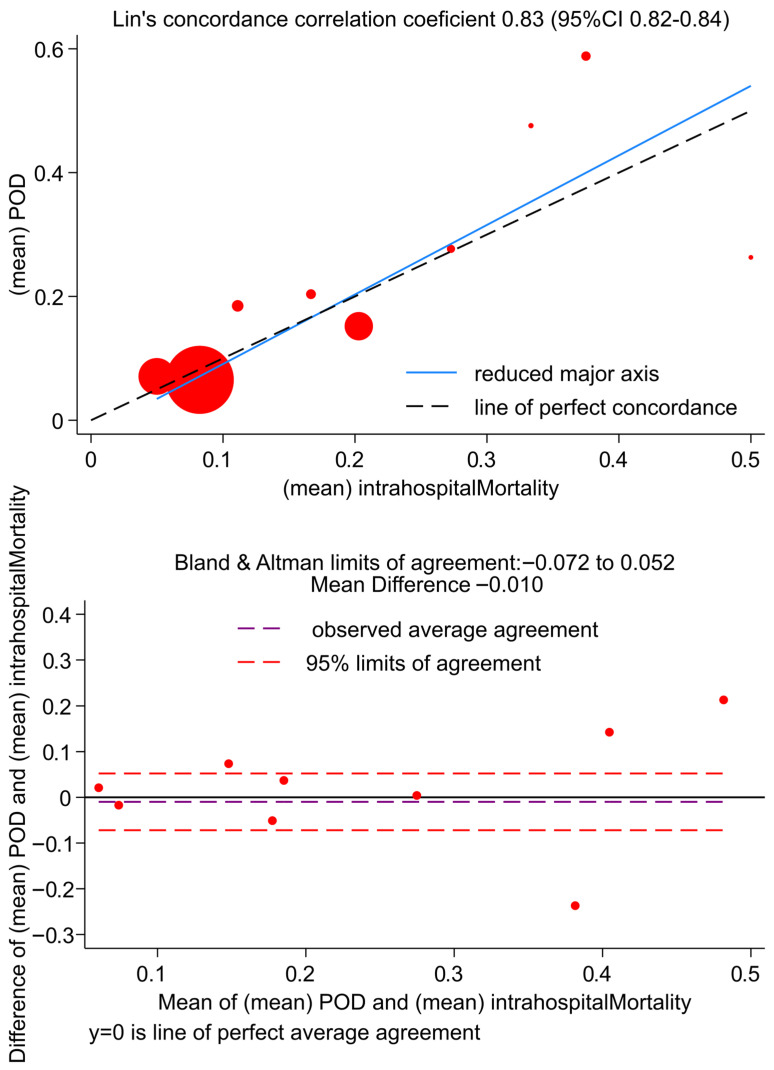
Concordance plots for observed and predicted intra-hospital mortality rates according to XISS groups. (**Upper panel**): Lin’s concordance correlation plot. (**Lower panel**): Bland and Altman limits of agreement.

As shown in Figure 7 and in Supplementary Table S1, TMPM POD and hospital mortality rates, assessed per XISS score strata, are in good agreement when the observed mortality is low; however, the agreement is questionable when mortality reaches values of about 40%.

Correspondingly, the Lin’s concordance correlation coefficient is good (Lin’s CCC 0.81, 95%CI 0.82–0.84), though not optimal (Figure 7, upper panel); at the Bland and Altman plot (Figure 7, lower panel) the limits of agreement are sufficiently small, but some significant correlation between the TMPM POD and hospital mortality difference and the mean are present (R = 0.216, *p* < 0.001).

## 4. Discussion

The PaTraME project demonstrates that leveraging routinely collected administrative discharge data (SDO) together with the STATA-derived XISS and TMPM-POD scores provides a low-cost, widely available toolkit for continuous surveillance of a trauma system over a large geographical area (the entire province of Pavia). In this study,

1. Case volume varied significantly between 2014 and 2021, with a steady increase until 2019, a marked drop during the COVID-19 pandemic in 2020, and a partial rebound in 2021; these fluctuations highlight the need for continuous monitoring and network readiness.
2. Severity distributions (XISS and TMPM-POD) were higher in patients admitted to the hub versus spoke centers, confirming the tool’s ability to reflect case complexity.
3. Centralization to the hub improved significantly, reflecting enhanced prehospital triage, Emergency Medical Services (EMS) training, and adherence to regional protocols. The regional trauma-network structure and EMS centralization framework remained formally stable throughout the study period, as defined by Lombardy Regional Decree n. 8531 (01/10/2012), including hospital designation (CTS/PST) within the provincial network. Hence, the observed improvement in centralization over time likely reflects system maturation and organizational learning (e.g., increased experience, strengthened coordination, and continuous training), rather than a change in formal protocols. Nevertheless, we acknowledge that external factors not captured in administrative data—such as demographic changes and local organizational refinements—may have influenced centralization patterns and should be considered when informing policy decisions.
4. Mortality (in-hospital, 30-day, and 180-day) showed no significant trends, confirming that increased centralization and stable patient volumes did not compromise overall outcomes, but rather maintained high-quality care.

The findings support TMPM-POD as a valid predictor of in-hospital mortality: average POD values showed good agreement with observed mortality at lower risk levels, while some discrepancies emerged when mortality approached higher values. Overall correlation remained high, indicating that TMPM-POD reliably tracks mortality trends, although less accurately at the upper risk spectrum. This pattern suggests a reduced calibration at the extreme end of predicted risk, which may limit TMPM-POD for individual-level prognostication in the highest-risk strata. However, PaTraME is primarily intended for system-level monitoring and benchmarking, where TMPM-POD supports risk adjustment of aggregated outcomes and longitudinal surveillance; therefore, deviations at the upper risk spectrum warrant cautious interpretation of extreme-risk bands, without undermining the framework’s utility for governance and quality monitoring.

These findings underscore several key advantages and future directions:

- Zero-cost scalability: Every hospital collects SDO for reimbursement; no dedicated registry infrastructure or manual coding is required beyond applying the free TMPM module in Stata. This makes the approach readily adoptable by regions or countries using ICD coding.
- Real-time monitoring and benchmarking: Health authorities can generate regular reports on case volumes, severity distributions, centralization, and mortality without incremental costs. Such reports enable identification of undertriage “hot spots,” seasonal surges, or gaps in EMS coverage.
- Policy and planning: Mapping centralization against EMS response times, spoke distribution, and hospital capacity, planners can optimize resource allocation (e.g., positioning of air-ambulance bases or adding training in peripheral EDs) to further improve hub access.
- Severity stratification: Clear differences in XISS and TMPM-POD between hub and spoke patients validate the discriminative power of the tool, highlighting spoke hospitals consistently receiving higher-severity cases than is safe for their resources.
- Identification of improvement margins: Although hub-admission has increased, 38.5% of major-trauma cases still presented to spoke centers—an undertriage rate that could be reduced. Moreover, spoke patients’ mean XISS, while lower than hub cases, remains relatively high for facilities with limited trauma resources, suggesting need for targeted training or restructuring.
- Extensibility to other outcomes: The same dataset can be analyzed for other quality indicators—such as splenectomy rates in blunt abdominal trauma, length of stay or distribution of interventions (e.g., angioembolization or surgical airway). Incorporating such metrics could build a comprehensive trauma performance dashboard.
- National and international benchmarking: Standardizing on XISS/TMPM-POD across all Italian provinces (or regions using ICD-9/10-CM) would enable direct comparisons of system performance, promoting best-practice sharing and quality improvement initiatives.

The ICD-9-CM-based monitoring tools should be viewed as complementary to traditional trauma registries rather than a replacement. Trauma registries provide higher clinical granularity (e.g., physiologic variables, prehospital triage, process-of-care indicators, complications, and functional outcomes), enabling detailed case-mix adjustment and patient-level quality improvement; however, they are resource-intensive and often limited by incomplete coverage, delayed reporting, and constrained scalability. In contrast, an administrative-data framework such as PaTraME leverages routinely collected discharge information to enable continuous, low-cost, population-based surveillance with timely feedback for governance and policy planning at the expense of reduced clinical detail. Consequently, PaTraME is best suited for system-level monitoring and benchmarking of longitudinal trends (volumes, centralization patterns, and risk-adjusted mortality), while registry-level data remain essential when the objective is patient-level prognostication and detailed performance improvement.

Looking forward, the transition from ICD-9-CM to ICD-10-CM will require adaptation of administrative-data severity modeling. Rather than relying solely on code crosswalks, a key future direction of PaTraME is to exploit linked administrative datasets to develop and validate data-driven models, including machine-learning approaches, that may achieve equal or improved performance compared with TMPM and can be trained directly on ICD-10-CM–based inputs. This strategy would allow the monitoring framework to remain scalable and comparable over time, while updating the underlying risk-stratification algorithm to the newer coding system and potentially improving discrimination and calibration at the upper risk spectrum.

This study has some limitations. First, the retrospective reliance on ICD-9-CM discharge codes inevitably introduces risk of misclassification, as coding errors or omissions can distort injury profiles and omit critical physiologic data such as blood pressure or Glasgow Coma Score [12,13]. We could not directly assess ICD-9-CM coding accuracy through chart review; therefore, differential coding practices across hospitals may influence ICD-derived severity estimates, although outcome-based agreement between TMPM-POD and observed mortality supports overall model reliability in this setting. Second, as SDO records lack rough prehospital data—EMS response times, on-scene interventions, and transport physiology—we cannot fully assess prehospital care impact. Third, patients transferred or diverted outside the province could not be fully captured at the receiving hospital level, potentially affecting centralization estimates. In addition, coding practices and granularity may differ between hub and spoke hospitals (particularly in peripheral centers), potentially leading to differential misclassification of injury profiles and affecting hub–spoke comparisons of XISS/TMPM-POD; nevertheless, this does not undermine PaTraME’s role as a system-level monitoring tool for longitudinal surveillance. Finally, our focus on mortality overlooks recovery and function data; without linkage to rehabilitation, claims, or patient-reported outcomes, we cannot comment on long-term disability, quality of life, or return to work.

Moving forward, linking SDO data with rehabilitation registries and patient-reported outcomes could broaden evaluation to functional recovery and long-term cost-effectiveness. Expanding beyond mortality to other quality indicators will enrich system monitoring. In the longer term, machine-learning models trained on these datasets may predict resource needs—ICU beds, operating theatre time, and blood products—and stratify patients at highest risk, guiding targeted interventions and continuous improvement across the trauma network.

By addressing these areas, the PaTraME framework can evolve into a comprehensive, scalable trauma-system observatory—supporting continuous quality improvement from the provincial to the national level [14].

## 5. Conclusions

The PaTraME project supports TMPM-POD as a valid predictor of observed in-hospital mortality and confirms that routinely collected administrative discharge data, when paired with the XISS and TMPM-POD severity scores, constitute a powerful, low-cost surveillance system for regional trauma networks. Over eight years in Pavia Province, this approach revealed a dynamic case volume, significantly improved primary centralization to the hub, and consistently maintained low mortality rates. Crucially, the clear separation of XISS and TMPM-POD distributions between hub and spoke hospitals not only validates the tool’s discriminative ability, but also pinpoints peripheral centers managing disproportionately severe cases, highlighting targets for enhanced training or resource allocation.

Because every hospital already generates SDO records, PaTraME is immediately scalable to larger regions or national health systems using ICD-9 coding. Beyond monitoring mortality, it can be extended to track splenectomy rates, imaging and intervention timelines, readmissions, and—through future data linkages—functional outcomes and cost-effectiveness. Embedding PaTraME within automated dashboards would empower health authorities to detect undertriage “hot spots”, optimize EMS deployment, and benchmark performance in real time.

In sum, PaTraME offers a pragmatic blueprint for continuous, system-wide quality improvement in trauma care: from validating centralization protocols today to guiding resource planning and patient-centered metrics tomorrow.

## Figures and Tables

**Table 1:** Traumatic patients epidemiology in Pavia Province in the period 2014–2021.

Characteristics	Median (IQR) N (%) N = 1959
Age (years)	77.0 (59–85) *
Gender (male)	1043 (55.7) *
XISS	17.0 (16–20)
TMPM-POD	0.05 (0.04–0.09)

* 287 missing values for age; 86 missing for gender.

**Table 2:** Trauma patients distribution per year and center type (negative binomial regression model LR Chi2 (8) = 3867, *p* < 0.001).

Variable	N (%)	IRR (95%CI) vs. Previous	*p*-Value *	Interaction of Year and Centre *p*-Value	Spoke N (%)	*p*-Value	Hub N (%)	*p*-Value *
**YEAR**			**<0.001**	**<0.001**		**<0.001**		**<0.001**
2014	232 (12)	1			93 (12)		139 (11)	
2015	250 (13)	1.07 (1.05–1.10)	0.073		104 (14)		146 (12)	
2016	255 (13)	1.02 (0.99–1.04)	0.811		110 (15)		145 (12)	
2017	246 (12)	0.97 (0.95–1.00)	0.022		97 (13)		149 (12)	
2018	249 (13)	1.00 (0.98–1.03)	1.000		114 (15)		135 (11)	
2019	304 (15)	1.25 (1.23–1.28)	<0.001		111 (15)		193 (16)	
2020	195 (10)	0.66 (0.64–0.68)	<0.001		63 (8)		132 (11)	
2021	228 (12)	1.22 (1.19–1.26)	<0.001		63 (8)		165 (14)	
**Centre Type**			**<0.001**		-	-	-	-
Spoke	755 (39)	1						
Hub	1204 (61)	1.56 (1.54–1.57)						

* post hoc comparisons with Bonferroni correction.

## Data Availability

The data presented in this study are available on request from the corresponding author. Because the data are not publicly available due to privacy.

## Supplementary Materials

The following supporting information can be downloaded at: https://www.mdpi.com/article/10.3390/medsci14010013/s1, Table S1: Mean TNPM-POD and Mortality rate over XISS score strata.

## Abbreviations

The following abbreviations are used in this manuscript:

ICD-9-CM	International Classification of Disease—9th revision—Clinical Modification
SDO	Scheda Dimissione Ospedaliera (Hospital Discharge Records)
GAT	Technical Study Group
CTS	Highly Specialized Trauma Center
CTZ	Zone Trauma Centre
PST	Trauma First Aid
SIAT	Integrated System for Trauma Patient Care
TMPM	Trauma Mortality Probability Model
POD	Probability of Death
PaTraME	Pavia Trauma Management Epidemiology
XISS	Extended Injury Severity Score
ED	Emergency Department
IRR	Incidence Rate Ratio
CI	Confidence Interval
