# Supplementary material for: Validation of an ICD-9-CM-Based Monitoring Tool for Regional Trauma Systems: The PaTraME Study in Pavia Province, Italy
Source: Med Sci (Basel). 2025 Dec 27;14(1):13. doi: 10.3390/medsci14010013 (PMC12821484; doi:10.3390/medsci14010013)
Supplement: Supplementary file 1 [file medsci-14-00013-s001.zip › medsci-4063624-supplementary.pdf]

Supplementary Table 1. Mean TNPM-POD and Mortality rate over XISS score strata

XISS Group	N	Mean TMPM-POD	Mortality Rate	Difference
15–19	1311	0.0653	0.0824	–0.0171
20–24	361	0.0709	0.0499	0.0210
25–29	207	0.1520	0.2029	–0.0509
30–34	27	0.1848	0.1111	0.0737
35–39	18	0.2037	0.1667	0.0370
40–44	11	0.2768	0.2727	0.0041
45–49	3	0.4759	0.3333	0.1426
50–54	2	0.2631	0.5000	–0.2369
55–59	0	—	—	—
60–64	0	—	—	—
65–69	0	—	—	—
70–74	0	—	—	—
75–79	16	0.5882	0.3750	0.2132
**Missing XISS**	**3**	—	0.0000	—
**Total**	**1959**			
